# Thyroid Gene Mutations in Pregnant and Breastfeeding Women Diagnosed With Transient Congenital Hypothyroidism: Implications for the Offspring’s Health

**DOI:** 10.3389/fendo.2021.679002

**Published:** 2021-10-14

**Authors:** Maria C. Opazo, Juan Carlos Rivera, Pablo A. Gonzalez, Susan M. Bueno, Alexis M. Kalergis, Claudia A. Riedel

**Affiliations:** ^1^ Millennium Institute on Immunology and Immunotherapy, Facultad de Ciencias de la Vida, Departamento de Ciencias Biológicas, Universidad Andres Bello, Santiago, Chile; ^2^ Instituto de Ciencias Naturales, Facultad de Medicina Veterinaria y Agronomía, Universidad de las Américas, Santiago, Chile; ^3^ Millennium Institute on Immunology and Immunotherapy, Departamento de Genética Molecular y Microbiología, Facultad de Ciencias Biológicas, Pontificia Universidad Católica de Chile, Santiago, Chile; ^4^ Departamento de Endocrinología, Facultad de Medicina, Escuela de Medicina, Pontificia Universidad Católica de Chile, Santiago, Chile

**Keywords:** offspring, pregnancy, breastfeeding, iodine, thyroid hormones, congenital hypothyroid women, genetic counseling

## Abstract

Fetus and infants require appropriate thyroid hormone levels and iodine during pregnancy and lactation. Nature endorses the mother to supply thyroid hormones to the fetus and iodine to the lactating infant. Genetic variations on thyroid proteins that cause dyshormonogenic congenital hypothyroidism could in pregnant and breastfeeding women impair the delivery of thyroid hormones and iodine to the offspring. The review discusses maternal genetic variations in thyroid proteins that, in the context of pregnancy and/or breastfeeding, could trigger thyroid hormone deficiency or iodide transport defect that will affect the proper development of the offspring.

## Introduction

Pregnancy and lactation are challenging physiological periods due to the mother supplies thyroid hormones and iodine to the fetus and infant, respectively. Given that, there is an increase on iodine and thyroid hormone requirements, a proper iodine status and thyroid hormone levels in pregnant and breastfeeding women must be guaranteed (1). Unfortunately, iodine insufficiency and thyroid hormone (TH) deficiency are frequent in pregnancy and breastfeeding (2). The statistics showed that 2.5% of pregnant and breastfeeding women consume insufficient iodine in their diet (3) and the prevalence of hypothyroidism is about 2%–3% in pregnant women (4). The progeny gestated and/or lactated under iodine deficiency or thyroid hormone deficiency, like hypothyroidism or hypothyroxinemia, is at higher risk of suffering irreversible cognitive impairment like attentional deficit, low intelligent quotient, and intellectual disability (5). Women carrying thyroid gene variations could be more sensitive to suffer thyroid hormone deficiency during pregnancy and/or iodide transport defect (ITD) during lactation. Special attention are those women that were diagnosed at birth with transient congenital hypothyroidism (TCH). TCH is a temporary deficiency of thyroid hormones identified after birth (6). TCH has several different causes like endemic iodine deficiency, iodine excess, maternal antithyroid medication, maternal antibodies, and genetics. This review will focus on thyroid gene variations that will risk the pregnant or lactating women to suffer thyroid hormone deficiency or ITD during pregnancy and/or lactation (7, 8). We will review these thyroid gene variations reported in the literature that caused dyshormonogenic TCH. The point of this review is to provide awareness to scientists and physicians to prevent thyroid hormone deficiency and ITD during pregnancy and lactation in those women diagnosed with dyshormonogenic TCH. These maternal genetic mutations on thyroid proteins could irreversibly damage the appropriate development of the offspring.

## Functional Maternal Thyroid Proteins Are Needed for Fetus Development

**Figure 1:**
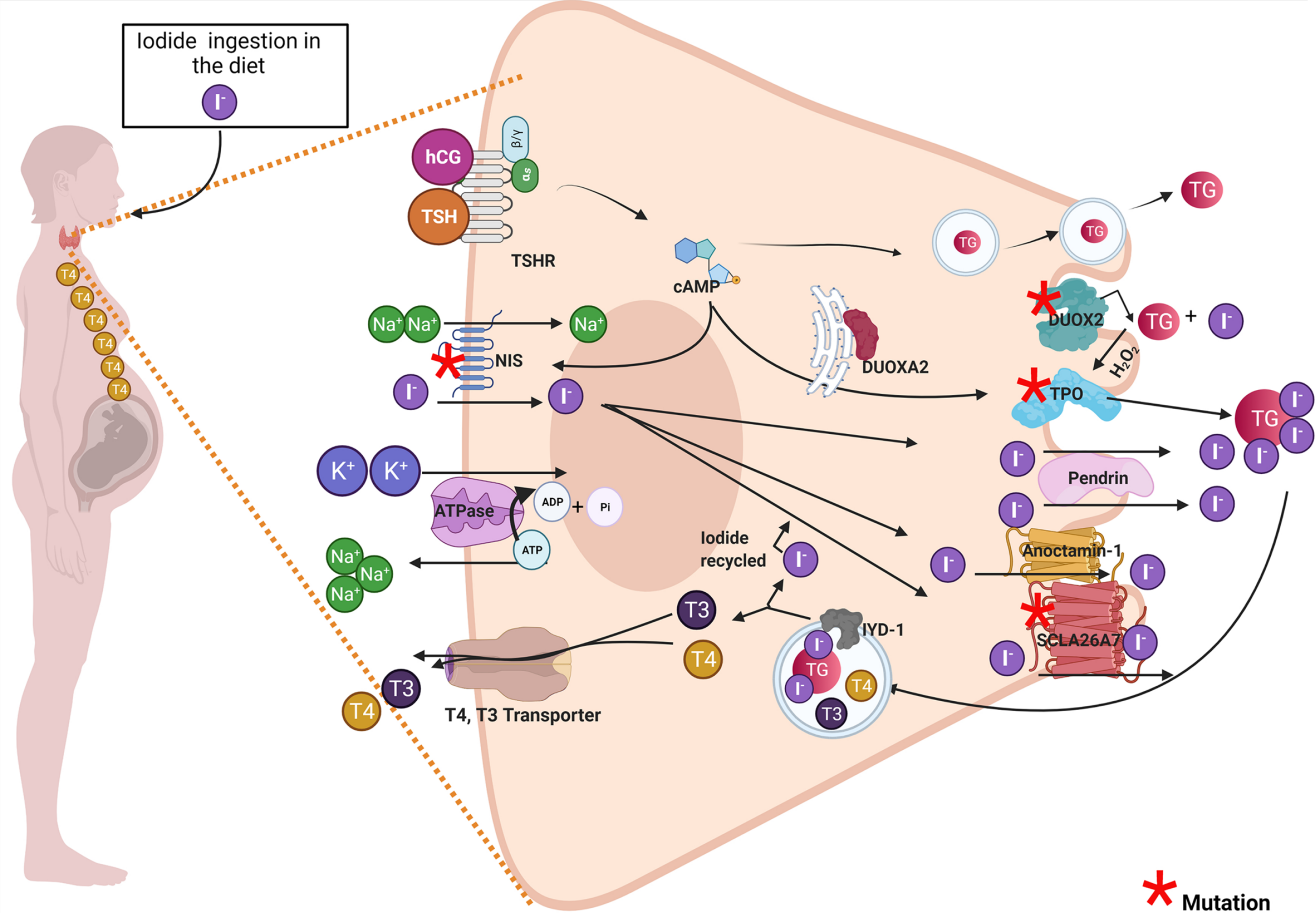
Pregnant woman diagnosed with transient congenital hypothyroidism due to mutations in thyroid proteins could develop thyroid hormone deficiency and impaired the offspring’s development. The drawing shows a thyroid cell of a pregnant woman and the principal thyroid proteins involved in thyroid hormonogenesis. Proteins with red asterisk have been associated in the literature with thyroid dyshormonogenesis in transient congenital hypothyroid patients. Among these proteins are DUOX2, DUOXA2, DUOX1, DUOXA1, TPO, NIS, and SLCA27 (see red asterisk). The maternal thyroid gland must synthesize thyroid hormones for mother and the fetus. The high demand for thyroid hormone synthesis in pregnancy could stress the thyroid gland especially in women carrying genetic variations on thyroid proteins and that were diagnosed with transient congenital hypothyroidism (TCH). These women diagnosed with TCH (carrying monogenic mutations in DUOX2 or DUOXA2 or digenic mutations in DUOX2 and DUOXA1 or mutations in TPO or NIS) that were euthyroid before pregnancy could develop thyroid hormone deficiency like hypothyroidism or hypothyroxinemia during pregnancy. Both hypothyroxinemia and hypothyroidism are thyroid hormone deficiency conditions that risk the proper fetus development and have deleterious consequences in the offspring.

In the first 5 months of pregnancy, the maternal thyroid gland will be challenged to supply THs to the fetus, especially T_4_ (9, 10). Thus, the mother will synthesize THs for her own needs and the fetus (**Figure 1**) (11, 12). This new scenery causes physiological stress to the maternal thyroid gland. To overcome this challenge, the pregnant woman should increase her daily iodine intake to 250 μg/day. The thyroid-stimulating hormone (TSH) and the human chorionic gonadotropin (hCG) will stimulate the maternal thyroid gland to produce T_4_ and T_3_ during the first trimester of pregnancy (13, 14). The hCG shares structural features with TSH (15). hCG and TSH will increase iodine uptake and thyroid hormone synthesis by binding to the TSH receptor (16). TSH and hCG will stimulate maternal thyroid gland growth and the expression of thyroid proteins like Na^+^/I^−^ symporter (NIS), pendrin (PDS), thyroglobulin (TG), thyroid peroxidase (TPO), dual oxidase 2 (DUOX2), and dual oxidase maturation factor 2 (DUOXA2) (17). The expression of NIS at the basolateral membrane of the thyrocyte will increase the uptake of iodide from the blood into the thyrocyte. Iodide will diffuse through the cytoplasm to the apical side of the thyrocyte, where transporters like PDS, anoctamin-1 (ANO1), and SLA26A7 will transport it to the colloid (18). The thyrocytes also express the cystic fibrosis transmembrane conductance regulator (CFTR) (18). CFTR transports chloride, and more studies are required to evaluate whether CFTR transports iodide. At the colloid, iodide will be oxidized and incorporated in the tyrosyl residues of TG by TPO (18). The organification of TG requires hydrogen peroxide (H_2_O_2_) which is supplied by dual oxidase system (DUOX). It has been described that two DUOX enzymes (DUOX1 and DUOX2) and their respective maturation factors (DUOXA1 and DUOXA2). These maturations factors reside at the endoplasmic reticulum, and they are essential for DUOX function (**Figure 1**). hCG and TSH will stimulate the endocytosis of iodinated TG. In the endo-lysosomes, TG will be digested by proteolytic enzymes like lysosomal dipeptidase, glutamate carboxypeptidase, and cathepsins B, K, L, and S releasing T_3_ and T_4_. THs will efflux the thyroid cell to the blood stream mainly by transporters like monocarboxylate transporter (MCT8) (19). It has been reported that certain genetic variations in thyroid proteins affects the function of the thyroid gland (20–22). There are mutations in thyroid genes that cause thyroid dyshormonogenesis, which is responsible for 10%–15% of congenital hypothyroidism (CH) (23). Newborns diagnosed with CH must be treated immediately with T_4_ to avoid neurological damage (23). Moreover, the etiology of CH should be determined by measurement of serum T_3_, T_4_, TSH, and TG and thyroid ultrasonography, thyroid scintigraphy, and perchlorate discharge test should be performed (23, 24). These biochemical and clinical analyses will reveal if the infant suffers transient congenital hypothyroidism (TCH) or permanent congenital hypothyroidism (PCH) (24). Those patients that have PCH will be kept for life with T_4_ treatment. Instead, thyroid function will be reassessed to those patients diagnosed with TCH at 3 years of age. If their thyroid function has been normalized, the T_4_ treatment will stop. This situation occurs for patients with TCH given that by progressing in age they require less thyroid hormone production related to their weight and the thyroid gland will be able to overcome the demands (23–26). *Moreno et al.* emphasize that it is crucial for those women that have been diagnosed with TCH due to monoallelic or biallelic mutations on thyroid proteins to be followed up during pregnancy for the risk of suffering hypothyroidism in this period and therefore will affect the appropriate development of the fetus (23, 27). Thyroid hormone synthesis can be impaired in pregnant women diagnosed at birth with TCH. Even though they were euthyroid before pregnancy, they could fall back in thyroid hormone deficiency due to expressing variants of thyroid protein that are in some degree functionally impaired (28). The thyroid gland of these women during pregnancy could be at risk of dyshormonogenesis endangering the proper development of the fetus. The recommendation of the Endo-European reference network (ERN) that patients with TCH should receive genetic testing to identify mutations causing thyroid dyshormonogenesis is of importance (23, 24). In the next section, the mutations in thyroid proteins that have been described to cause TCH will be discussed.

## Variations in Maternal Thyroid Genes That Could Impair the Development of the Fetus

It has been described in the literature that variations on thyroid proteins like NIS, DUOX2, DUOXA2, TPO and Tg are responsible for TCH. In this section we briefly discuss the relevance of of these proteins variations in TCH women when they reached pregnancy. The NIS mutants could induce ITD causing TCH or PCH (29). However, the severity and the onset of the disease phenotype will depend on the type of NIS mutation, if it is monoallelic or biallelic, the period of life, and iodide ingestion (30, 31). Therefore, physicians should prevent thyroid hormone deficiency in pregnant women diagnosed with TCH carrying NIS mutations (**Figure 1**). There are other transporters expressed in the thyroid cells like PDS, ANO-1, SLC26A7, and CFTR (32–34). PDS, ANO-1, and SLC26A7 are localized at the apical side of the thyrocyte, and their physiological role have been associated with iodide efflux into the colloid (35). Mutations on *SLC26A4* that encodes PDS has been associated with Pendred syndrome, characterized by congenital bilateral sensorineural hearing loss and in childhood can appear diffuse or multinodular goiter (36). Most of the patients with Pendred syndrome are euthyroid; however, their thyroid function can be impaired if they are exposed to low iodine intake (37). ANO-1 and CFTR variants have not been associated with TCH. A study performed in Saudi Arabia, by using whole-exome sequencing (WES) described mutations in SLC26A7 that could be responsible in part for CH (38). Another thyroid protein variants that have been reported to be responsible for TCH are monoallelic and biallelic mutations of DUOX2 (27, 39, 40). DUOX2 is a transmembrane protein localized at the apical side of the cell, and it belongs to (NADPH)-oxidase family whose function is involved in H_2_O_2_ generation (**Figure 1**). H_2_O_2_ is required for thyroid hormone synthesis specifically for iodide organification in tyrosyl residues of TG by TPO (**Figure 1**) (35). The prevalence of *DUOX2* mutations is highly variable in the population. From 2019, approximately 105 *DUOX2* mutations are described. Some of them were in-frame deletions, missense, nonsense, splice site, and frameshift mutations (41, 42). Moreover, mutations in the gene *DUOXA2* were also reported in TCH patients (43). DUOXA2 is an endoplasmic reticulum transmembrane protein that helps DUOX2 mature through the ER and translocate to the plasma membrane (44). Zamproni et al. found a biallelic mutation in the *DUOXA2* gene that leads to a partial iodide organification defect (45) and Liu et al. found a monoallelic missense mutation in DUOXA2 that causes mild TCH (46). Besides, DUOX2 and DUOXA2 thyrocytes also express DUOX1 and DUOXA1. DUOX1 has 83% protein sequence homology with DUOX2; however, its expression is lesser than DUOX2 and its function in the thyroid gland is not clear yet as DUOX1 mutations do not cause CH (47). Aycan et al. described two patients with severe CH that have biallelic mutations in DUOX2 and DUOX1, suggesting that DUOX1 can replace the function of DUOX2 when this enzyme is not available (48). DUOXA1 in similar fashion as DUOXA2 helps DUOX1 to mature through the ER and mutations in DUOXA1 has not been associated yet with CH (47). The possible implication of DUOX1 and DUOXA1 in CH is under intense debate, as the related mutations were reported to be always associated with DUOX2 or DUOXA2 mutants which are the principal proteins involved in the thyroid H_2_O_2_-generating system. Interestingly, Wang et al. sequence 16 genes related with CH by next-generation sequence (NGS) in 377 CH patients and found patients with biallelic mutations in DUOX2 and DUOXA1, suggesting that DUOX1 and DUOXA1 could also play a role in thyroid hormone synthesis (49). TPO is responsible for iodide organification and the coupling of tyrosyl residues, essential steps in thyroid hormone synthesis (50). Thus, the expression of TPO variants will severely affect thyroid hormone synthesis (37). In fact, it has been reported that TPO biallelic mutations caused PCH (51). Another study, performed in 243 Russian patients also showed an increased number of variants identified in thyroid dyshormonogenesis-associated genes mostly of the variants found in the *TPO* gene (52). It has been shown that certain patients with monoallelic mutations in *TPO* could develop PCH (53, 54), mild hypothyroidism (55), and TCH (56). It has been reported that there are 229 mutations in *TG*, the precursor of thyroid hormones (57). Some of them occur as biallelic or monoallelic and some are found inherited as monogenic or polygenic (57). A targeted NGS study performed in 19 patients mostly from France with CH due to dyshormonogenesis showed that TG was a site with the higher number of identified mutations followed by DUOXA2 (58). Interestingly, mutations in *TG* cause euthyroidism to mild or severe CH (57). Variants in iodotyrosine deiodinase (IYD) cause dyshormonogenic PCH, and there are no monoallelic mutations associated to TCH (57). Molecular biology techniques, like NGS and WES will help to understand the genetic component of CH. These molecular techniques like NGS and WES will provide data to fill the complex analysis of gene panels aiming to understand the mechanisms underlying disease development (59). Genetic counseling is recommended for women previously diagnosed with TCH to prevent thyroid hormone deficiency during gestation, and it is highly recommendable to follow thyroid function during pregnancy.

## Proper Iodide Nutrition Is an Essential Requirement for Infant Thyroid Hormone Synthesis

**Figure 2:**
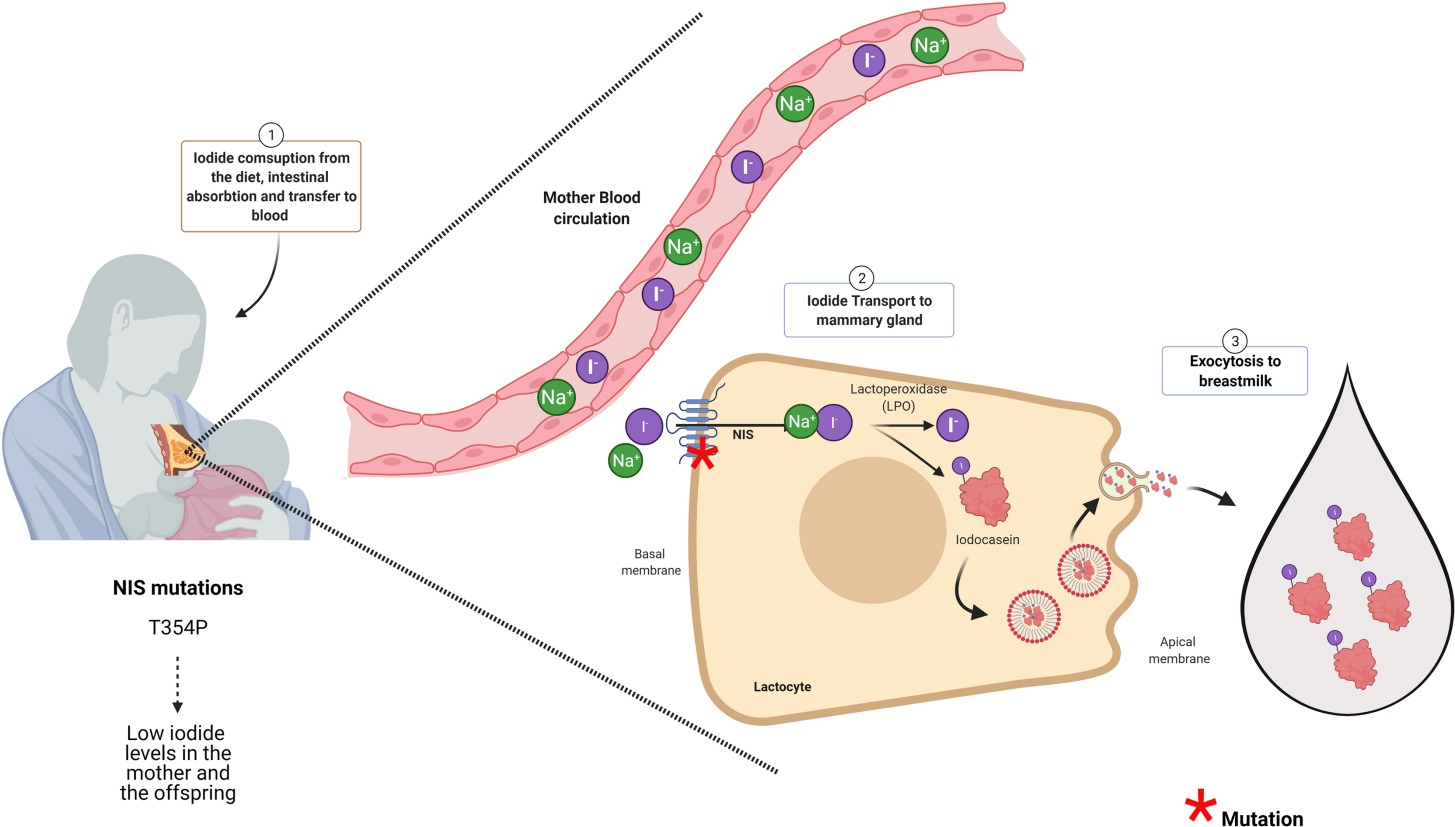
NIS and LPO are essential proteins for iodide accumulation in breast milk. The infant synthesizes its own thyroid hormones. For that, the mother should consume enough iodine to supply her baby with this micronutrient through the breast milk. The Na^+^/I^−^ symporter (NIS) at the intestine will transport iodide into the blood NIS expressed at the lactocytes will transport iodide from the blood to inside these cells. NIS in the lactocytes is located at the basal membrane. The iodide inside the lactocyte is incorporated into casein by the action of lactoperoxidase (LPO). Iodocasein molecules will be aggregate inside micelles. The micelles will fuse with the apical membrane to release their content by exocytosis to breast milk. Woman diagnosed with congenital hypothyroidism (CH) carrying NIS mutation (like T354P) accumulated low iodide in the milk even though she was under levothyroxine (LT_4_) treatment. Thus, women carrying NIS mutants that caused congenital hypothyroidism besides T_4_ treatment should receive iodide supplementation (66). Mutations in proteins with red asterisk have been associated in the literature with low iodide in breast milk as it is for NIS (see red asterisk). Even though, LPO is an essential enzyme for iodide presence in the milk, there are no reports in the literature indicating that mutations in LPO affects iodide content in breast milk.

Neonates and lactating infants require thyroid hormones for proper physical and neurological development. Even though there are traces of maternal thyroid hormones in the milk, the main source of these hormones for the infant derives from their own thyroid gland (60, 61). Thus, neonates and infants are required to ingest iodine for thyroid hormone synthesis (25). The iodine demand for the newborn is higher than an adult if we ponder their weight (62). Newborns, until 5 months of age need 15 µg/kg/day of iodine, and infants are required 90 µg/day of iodine (63). The newborn thyroid gland has a small iodine reserve close to 100 µg. Therefore, by considering their high demand for this micronutrient, the infants should have a daily intake of iodine (64). Breast milk is an excellent source of iodine; however, its content can vary from 5.4 to 2.170 μg/L (26, 65). Physiologically, iodine content in breast milk will depend mainly on the mother’s iodine consumption being influenced by iodization programs (25, 40). According to the WHO/UNICEF and the International Council for Control of Iodine Deficiency Disorders (ICCIDD), the recommended iodine intake for lactating women is 250 μg/day to ensure 100–150 µg of iodine/dl in the breast milk (26). Iodine is accumulated into the mother’s milk in the form of iodide, and this transport is mediated by NIS that is expressed in the mammary gland physiologically only during lactation (**Figure 2**) (67). NIS transports iodide against its electrochemical gradient from the blood into lactocytes, and its expression is regulated by oxytocin and prolactin (67). Other transporters like CFTR and ANO-1, that efflux iodide at the thyrocyte, are also expressed in the mammary gland (68, 69). Prolactin also regulates the expression of PDS, an apical thyroid iodide transporter, in lactocytes during lactation (70). The role of these transporters in iodide transport in the mammary gland must yet be elucidated. Iodide inside the lactocytes is oxidized and incorporated into casein by a lactoperoxidase (LPO), forming iodocasein molecules (**Figure 2**). These iodocasein molecules are aggregated inside micelles and will be secreted into breast milk (**Figure 2**) (71). The concentration of iodide in breast milk depends on environmental and genetic factors. Women living in regions with insufficient iodine intake are at risk of iodine deficiency during lactation (72). Among the environmental factors are the mother’s diet and the ingestion of thiocyanate and perchlorate (73–75). Perchlorate and thiocyanate are NIS inhibitors of iodide transport (29, 76, 77). Perchlorate is a competitive inhibitor of iodide; Llorente-Esteban et al. showed that the tiny perchlorate addition increases NIS K_m_ for iodide impairing thyroid hormone synthesis (78). Thiocyanate is a potent NIS inhibitor, and it has been described as a noncompetitive inhibitor (76, 79, 80). Therefore, the presence of these molecules in the mother’s diet will reduce the iodide uptake in lactocytes, diminish the concentration of iodide in breast milk, and reduce thyroid hormone synthesis in the infants affecting their development (81). Concerning genetic factors, particular attention should be taken with congenital hypothyroid women because they can carry NIS or LPO variants that impair the accumulation of iodide in breast milk during lactation. In the next section, NIS variants will be discussed.

## NIS Variants That Affect Iodide Concentration in Breast Milk

NIS is the principal molecule responsible for iodide uptake in the mammary gland playing a pivotal role in iodide concentration in breast milk (67, 82) (**Figure 2**). Eighteen NIS mutations have been reported in the *Slc5a5*, the gene that encodes for *NIS* (30, 83, 84). The type of mutations in *Slc5a5* includes nonsense, alternative splicing, frameshift, deletion, and missense, and they are responsible for causing ITD (20). ITD occurs as autosomal recessive disorder that results in CH (29). It has been reported that the severity and the onset of the CH varies and depends on the type of NIS mutation and iodide ingestion (30, 31). An important issue to consider is the situation of breastfeeding women diagnosed with CH bearing NIS mutation and treated with levothyroxine (LT_4_) (84). Even though LT_4_ will protect the mother from suffering hypothyroidism, their lactating offspring will not receive enough iodide, given that NIS is the only iodide transporter described in the mammary gland so far. PDS is also expressed at the mammary gland, its expression is stimulated by prolactin; however, PDS mutations affecting iodine content in breast milk has not been described (85). Regarding the NIS variants, there is only one report by Mizokami et al., showing ITD at the mammary gland during lactation (66). Mizokami et al. reported the case of a woman diagnosed with CH that received LT_4_ treatment (150 µg/day of LT_4_) and had low levels of iodide in her breast milk (54 µg/L) (66). Iodine supplementation was given to the mother to reach the minimum level of 90 µg/L of iodide in milk. This amount was accepted as sufficient to fulfill the newborn iodine necessities (66). The breastfeeding woman carried a missense variation in the *slc5a5* gene previously described by Fujiwara et al. This variant has a cytosine instead of adenosine in codon 354, leading to an exchange of threonine for proline (T354P) in NIS protein (86). NIS T354 mutant has lost function and is highly prevalent in the Japanese population (87). Regarding LPO, an important enzyme for iodide accumulation in breast milk, there are no specific mutations described yet in this protein that affects iodide content in breast milk.

## Discussion and Concluding Remarks

It is well established that patients born with PCH should be immediately treated with LT_4_ for life. However, those patients with TCH will stop LT_4_ treatment at a certain age when their thyroid function normalizes and present normal TSH and fT_4_, in fact most TCH patients are euthyroid at adulthood. Besides that, genetic counseling is highly recommended especially for women. Genetic counseling will allow to identify the mutations that are responsible for thyroid dyshormonogenesis. As we discussed in this review, proper maternal thyroid function in pregnancy is essential for appropriate fetus development. The requirement for thyroid function during pregnancy is higher in pregnant women than for nonpregnant women. Pregnancy requires increasing iodine consumption and the demand for functional thyroid proteins. The identification of mutations in thyroid proteins will help physicians to protect the women during pregnancy to suffer hypothyroidism or hypothyroxinemia. Women carrying genetic mutations on *DUOX*2, *DUOXA2*, TPO, or *NIS* gene, they must receive prophylactic LT_4_ treatment during pregnancy due to the higher probability to develop thyroid hormone deficiency during this period. The relevance of NIS mutants surpasses pregnancy, given they are also significant for infant development during breastfeeding. If the CH woman carries mutations of NIS protein, besides T_4_ treatment, she must receive prophylactic iodine supplementation during breastfeeding. Given that T_4_ treatment will only rescue the mother from hypothyroidism and not his/her lactating infant for thyroid hormone deficiency, medical doctors should care to supply the mother with enough iodide. One approach was described by Mizokami et al. The authors administered iodide to the lactating woman that carries NIS T354P mutation and follows the measurement of breast milk iodine concentration (BMIC) (66). This action restored the normal levels of iodide and protected the neonate from suffering the health consequences of hypothyroidism (66, 85). There are certain NIS variants that cause severe CH, and in these cases, mother supplementation with iodide will not rescue the lactating infant. In these cases, it will be highly recommendable to directly supplement the baby by giving him/her iodine through baby formula (63). Baby formula should have 10–60 µg of iodine/100 kcal or 5–75 µg of iodine/100 kcal according to the regulations of the Commission of European communities (88) and the US Food and Drug Administration (FDA), respectively (89).

Genetic counseling is essential for CH women to determine if they will need iodide supplementation during breastfeeding to ensure the proper development of their infant. LPO mutants had not yet been described in the literature, and they have not even been related to low iodide levels in breast milk. Based on the physiological relevance of iodide for the infant’s proper development, we highly recommend measuring its content in breast milk.

## Conclusions

The concluding remark of this mini review is to highly recommend women that have been diagnosed with dyshormonogenic CH to counsel for genetic study before pregnancy, aiming to determine which thyroid gene/s is or are mutated. By using this strategy, medical doctors could design the prophylactic treatment by giving the patient LT_4_ and/or iodide. This information will protect the offspring during gestation and breastfeeding from cognitive impairment. The mother is the only source of thyroid hormones at the early pregnancy and the source of iodide during breastfeeding. Women diagnosed with TCH who bear mutations in *DUOX2*, *DUOXA2*, *TPO*, *TG*, *SLC26A7*, or *NIS* genes and are euthyroid before pregnancy should be prophylactic treated with LT_4_ during pregnancy, aiming to have appropriate thyroid hormone level for the development of the fetus. Although women who carry mutations for NIS are treated with LT_4_ during lactation, they must increase the consumption of iodide, and BMCI should be monitored. The fact that there is only a single report in the literature associating that a lactating woman carrying a NIS mutant should receive iodine supplementation during lactation to protect the development of the lactating infant, increases the alarm for gynecologist and endocrinologist to evaluate for NIS variations in women diagnosed with TID. Moreover, it is necessary to encourage NGS and WES analyses in patients diagnosed with TCH due to dyshormonogenesis. These techniques will unveil the complexity of thyroid genes that are required for the proper function of thyroid gland for the human health protection.

## Author Contributions

MO and CR worked: All authors have made a substantial, direct, and intellectual contribution to the work and approved it for publication. All authors have read and agreed to the published version of the manuscript. All authors contributed to the article and approved the submitted version.

## Funding

This work was supported by FONDECYT 11180739 (MO), 1191300 (CR), Millennium Institute on Immunology and Immunotherapy (P09/016-F and ICN09_016) (MO, CR), and Nucleus project DI-03-19/N 303 (CR and MO).

## Conflict of Interest

The authors declare that the research was conducted in the absence of any commercial or financial relationships that could be construed as a potential conflict of interest.

## Publisher’s Note

All claims expressed in this article are solely those of the authors and do not necessarily represent those of their affiliated organizations, or those of the publisher, the editors and the reviewers. Any product that may be evaluated in this article, or claim that may be made by its manufacturer, is not guaranteed or endorsed by the publisher.
